# Isolation of a Highly Efficient Antigenic-Protein-Degrading *Bacillus amyloliquefaciens* and Assessment of Its Safety

**DOI:** 10.3390/ani10071144

**Published:** 2020-07-06

**Authors:** Yang Li, Baozhu Guo, Chong Li, Weiwei Wang, Zhengke Wu, Guohua Liu, Huiyi Cai

**Affiliations:** The Key Laboratory of Feed Biotechnology of Ministry of Agriculture, National Engineering Research Center of Biological Feed, Feed Research Institute, Chinese Academy of Agricultural Sciences, No.12 Zhongguancun South Street, Haidian District, Beijing 100081, China; liyang8906@163.com (Y.L.); guobaozhu957@163.com (B.G.); lichong@caas.cn (C.L.); dbnywzw@163.com (W.W.); wzk199107@163.com (Z.W.); liuguohua@caas.cn (G.L.)

**Keywords:** *B. amyloliquefaciens*, fermentation, soybean meal, antigenic protein, safety assessment

## Abstract

**Simple Summary:**

Soybean meal (SBM), a byproduct of soybean oil extraction, is a commonly used dietary protein in the poultry and swine feed industries because of its high quality protein and relatively well-balanced amino acids. However, major antigenic proteins in SBM, glycinin and β-conglycinin, can trigger allergic reactions, including intestine villus atrophy and other malabsorption syndromes, in newborn animals. Microbial fermentation is considered an economically viable processing technique to reduce the content of antigenic proteins, and improve the nutritional quality of SBM. The kind of microorganism used in fermentation is one of the major factors affecting the nutritional value of SBM. In this study, a highly efficient *Bacillus. amyloliquefaciens* strain was successfully isolated with convenient and effective plate tests, and used in a fermentation experiment. Fermentation with *B. amyloliquefaciens* for 24 h effectively degraded the glycinin and β-conglycinin in SBM, significantly improved the crude protein content and acid soluble protein concentration, and increased the total amino acid content. Furthermore, *B. amyloliquefaciens* had no adverse effects on animal health. These results indicate that the *B. amyloliquefaciens* strain isolated in this study is safe for animal use and can be widely used in SBM fermentation.

**Abstract:**

The aims of this study were to screen and isolate a highly efficient strain from the rumen of a cow that can degrade the antigenic soy proteins in soybean meal (SBM) and improve the nutritional value of SBM by fermenting it with this strain. The safety of this strain was investigated with an acute oral toxicity test. A *Bacillus amyloliquefaciens* strain was successfully screened with plate tests and fermentation. After solid state fermentation of SBM with B. *amyloliquefaciens* for 24 h, the amounts of glycinin and β-conglycinin, two major antigenic proteins in SBM, decreased by 92.32% and 85.05%, respectively. The crude protein content in the fermented soybean meal (FSBM) increased by 17.54% compared with that in SBM. Notably, the trichloroacetic-acid-soluble protein (TCA-SP) content, particularly small peptides and free amino acids, was 9.97-fold higher in FSBM than in SBM. The in vitro dry matter digestibility and digestible energy of SBM increased from 62.91% to 72.52% and from 10.42 MJ/kg to 13.37 MJ/kg (dry matter basis), respectively, after fermentation. The acute oral toxicity test suggested that the strain exerted no harmful effects on the relative organ weights, the morphological tissue structure, or the health of mice. These results indicate that the *B. amyloliquefaciens* strain isolated in this study is a safe strain for animals, and could be used to improve the nutritional quality of SBM by solid-state fermentation.

## 1. Introduction

Soybean meal (SBM), a co-product of soybean oil extraction from soybeans, is commonly used in the poultry and swine industries because of its high quality protein and relatively well-balanced amino acid content [1]. However, a variety of antinutritional factors (ANFs) in SBM, such as oligosaccharides, lectin, and allergenic antigens, hinder the digestion and absorption of its nutrients by animals [2,3,4]. Glycinin and β-conglycinin are the two main types of allergenic soybean proteins, accounting for about 30% and 40% of the total protein in SBM, respectively [5]. Previous studies have demonstrated that glycinin damages the intestinal morphology, with effects such as intestinal atrophy and necrosis, and causes malabsorption syndrome in animals [6,7,8]. β-Conglycinin causes hypersensitive immune response and negatively affects the growth performance of animals [9,10]. These drawbacks limit the extensive use of SBM in animal feeds. Therefore, the development of processing techniques that remove the harmful and antinutritional factors of SBM may produce a high-quality protein with functional properties [11].

Fermentation is considered an economically viable processing technique in which microorganisms are used to increase the protein concentration, reduce the content of ANFs, and enhance the nutritional value of SBM [12]. It has been documented that the apparent ileal digestibility of the dry matter, nitrogen and energy in fermented soybean meal (FSBM) is similar to that of fish meal [13]. Bacteria and fungi are the two main types of microorganisms used to ferment animal feed or feed ingredients. *Bacillus* spp. have a stronger capacity to resist heat and acid [14], and secrete many kinds of extracellular enzymes, including non-starch polysacchrides degrading enzymes and proteinase, and are therefore commonly used in bacteria-based fermentation [15]. Seo and Cho reported that the contents of glycinin and β-conglycinin in SBM decreased by 70% and 50%, respectively, after fermentation with *B. subtilis* KCCM11438P for 24 h [16]. Teng et al. reported that fermentation of SBM with *B. subtilis* SB102 degraded its large antigenic proteins, and increased the small-size proteins and the contents of some essential amino acids compared with those in unfermented SBM [17]. In addition to *Bacillus* spp., *Lactobacillus* spp. are also frequently used in solid state fermentation to produce organic acids and reduce the pH of SBM. Chi and Cho demonstrated that *Lactobacillus* spp. significantly reduced the pH of SBM, but did not decompose protein-based ANFs other than trypsin inhibitors after solid state fermentation for 36 h [18]. Fungal fermentation with several species of the genus *Aspergillus*, such as *A*. *niger* and *A*. *oryzae,* caused much smaller increases in small proteins and smaller reductions in larger antigenic proteins in SBM than bacterial fermentation [15]. The efficiency of fermentation in improving nutritional value of SBM depends on growth rate, extracellular enzymes and its activities of microorganisms involved [18]. Therefore, it is vital to choose a highly efficient allergen-degrading microorganism for the production of FSBM with high nutritional quality.

The glycinin and β-conglycinin in SBM does not cause hypersensitive immune response in gastrointestinal tract or affect the growth performance of ruminants as they do in pre-ruminant animals and nonruminant animals because the rumen contains a mixed community of microbes [19] that can inactivate and degrade glycinin and β-conglycinin [20]. About 12% to 38% of the total bacterial population in rumen are strains with proteolytic activities [21], so the rumen is an ideal place to select an appropriate bacterium for the decomposition of glycinin and β-conglycinin in SBM. In previous studies, target bacteria were successfully isolated from a preserved vegetable [22], traditional fermented soy foods [23], and animal intestinal tracts [24]. However, to our knowledge, no research has used a strain derived from the rumen flora to ferment SBM. The selected bacteria have previously been used to ferment SBM directly, and animal experiments have been conducted without considering the safety of the strain in these experiments. However, some bacteria may produce hemolysin, which is considered a virulence-related factor that impairs the intestinal epithelial cells [25,26]. Therefore, the objective of this study was to screen a highly efficient bacterial strain in a cow rumen with which to degrade the antigenic proteins in SBM, and to improve the nutritional quality of SBM by fermentation with this strain. The safety of this bacterium was evaluated with an acute oral toxicity test.

## 2. Materials and Methods

### 2.1. Isolation of Bacterial Strain

Rumen liquid was collected from a fistulated cow feeding at the Experimental Nankou Base of Chinese Academy of Agricultural Science (Beijing, China), and was filtered through double cheesecloth. An aliquot (1 mL) of the rumen liquid was homogenized in 9 mL of 8.5% (*w*/*v*) sterile salt water, and then incubated at 80 °C for 20 min to destroy other microbial cell [23,27]. Defatted milk agar pates were inoculated with this homogenate for bacterial culture by gradient dilution (10^−2^–10^−7^). These inoculated plates were cultured in a biochemical incubator at 37 °C for 24 h. After incubation, strains were selected according to the size of the transparent zone around colonies (data not shown). Six strains were isolated for a further screening experiment, and denoted ´GYL’. Two strains of *B. subtilis* stored previously in our laboratory, designated BSD2 and CICC21237, were used as the positive controls.

### 2.2. SBM and Medium Preparation

SBM was purchased from the Hopefull Grain & Oil Co. Ltd. (Langfang, Hebei, China). Agar plates containing antigenic protein (glycinin or β-conglycinin), starch, or xylan were used to determine the protease, amylase, and xylanase activities of the strains, respectively. The antigenic proteins were extracted from SBM according to the method reported by Liu et al. [28] and Deak et al. [29], with minor modification. Briefly, about 15 g of defatted soy flour was extracted with 0.03 M Tris-HCl (pH 8.5) in a 15:1 (*w*/*v*) buffer-to-flour ratio. The resulting slurry was stirred 200 r/min for 1 h at 45 °C and centrifuged at 9000× *g* for 40 min at 4 °C. After centrifugation, the supernatant was discarded and the precipitate was extracted two more times. The appropriate amount of solid NaHSO_3_ was added to the supernatant to achieve a concentration of 10 mM NaHSO_3_ and the pH was adjusted to 6.4 with 2 M HCl. The slurry was stored at 4 °C overnight and then centrifuged at 6500× *g* for 20 min at 4 °C. The glycinin-rich fraction was thus obtained. Solid NaCl was added to the supernatant to a final concentration of 0.25 M and the pH adjusted to 5.5 with 2 M HCl. The slurry stirred at 45 °C and 200 r/min for 30 min, and then centrifuged at 9000× *g* for 40 min at 4 °C. The supernatant was diluted two-fold with pure water and adjusted to pH 4.8 with 2 M HCl. The β-conglycinin-rich fraction was obtained after centrifugation at 6500× *g* for 20 min at 4 °C. The total precipitate was dissolved in pure water to prepare protein plates. Starch agar plates were prepared by adding 1 g of corn starch and 1.5 g of agar to 100 mL of Luria-Bertani (LB) medium (1% tryptone, 1% NaCl, and 0.5% yeast extract). Xylan agar plates were prepared by mixing 0.3 g of xylan and 1.5 g of agar to 100 mL of LB. To prepare defatted milk agar plates, 5 g of defatted milk (Yili, Inner Mongolia, China) and 1.5 g of agar were added to 100 mL of LB. All plates were autoclaved for 21min at 121 °C.

### 2.3. Solid State Fermentation

The SBM for fermentation was sieved through a 10 mesh screen. The inocula of eight bacteria were prepared by incubating a single colony in LB medium at 37 °C for 21 h. SBM (20 g) was mixed with distilled water to 50% (*w*/*v*) in a 150 mL conical flask and inoculated with fresh inoculum in a 5% ratio (*w*/*v*). Each mixture was incubated at 37 °C for 24 h, each treatment was performed in triplicate. After fermentation, the FSBM samples were dried at 60 °C for 4 h, ground, and passed through a 60-mesh screen. The fermented powder samples were stored at 4 °C before analysis.

### 2.4. Chemical Composition of FSBM

The crude protein content was estimated with a combustion analyzer (Dumatherm, Gerhardt, Germany) using ethylenediaminetetraacetic acid as the calibration standard. The content of trichloroacetic acid-soluble protein (TCA-SP) in SBM or FSBM was measured with trichloroacetic acid (TCA) according to the China National Standard (GB/T 22492-2008). Briefly, 1 g of sample was mixed with 20 mL of 15% (*w*/*v*) TCA, and the slurry was stirred at room temperature for 5 min and centrifuged at 4000× *g* for 10 min. The supernatant was collected, and the nitrogen content of the supernatant was determined with an automatic Kjeldahl Nitrogen Analyzer (FOSS, Hillerod, Denmark).

The contents of glycinin and β-conglycinin in the SBM or FSBM were analyzed using competitive enzyme-linked immunosorbent assay (ELISA) kits (Longkefangzhou Bio-Engineering Technology Company, Beijing, China). The total amino acid content was extracted with 6 M HCl and measured with an automatic amino acid analyzer (Hitachi L-8900, Tokyo, Japan).

### 2.5. Protein Extract and SDS-PAGE

The soluble proteins in SBM and FSBM were extracted as previously described by Yang et al. [1]. The soluble protein concentrations were quantified with Bio-Rad Protein Assay Kit (Bio-Rad, Hercules, CA, USA), according to the manufacturer’s protocol. The fractions of soluble protein in the SBM and FSBM were detected with 12% polyacrylamide separating gels containing 0.1% SDS in Tris-glycine buffer. About 15 μL of total denatured soluble protein sample was loaded into a well. The extracted soluble protein sample was concentrated at 80 V for 30 min and separated at 120 V for 60 min. The gel was stained with Coomassie Brilliant Blue R-250 (Bio-Rad) for 45 min, and destained with 7% (*v*/*v*) acetic acid.

### 2.6. Biochemical Characteristics and Molecular Identification of the Candidate Strain

The biochemical characteristics of the strain GYL6 were determined using a commercial kit (Hopebio Co., Ltd., Qingdao, China) according to the manufacturer’s instructions. The genomic DNA of strain GYL6 was extracted with the Ezup Genomic DNA Extraction Kit (Sangon Biotech Co., Ltd., Shanghai, China), according to the manufacturer’s protocol. The full-length sequence of 16S rRNA of strain GYL6 was amplified by polymerase chain reaction (PCR) with the primers 27F (5′-AGAGTTTGATCMTGGCTCAG-3′) and 1492R (5′-TAGGG TTACCTTACGACTT-3′) (Sangon Biotech Co., Ltd.). The gyrase B (gyrB) gene of strain GYL6 was amplified with PCR using primers UP1F (5′-GAAGTCATCATGACCGTTCTGCA-3′) and UP2R (5′-AGCAGGGTACGGATGTGC GAGCC-3′) (Sangon Biotech Co., Ltd.). The PCR amplification was carried out in a 25 μL reaction mixture containing 25 ng of template DNA, 0.5 μL of each primer, 1 μL PCR premix, 0.2 μL Taq mix, 2.5 μL buffer and PCR-grade water to adjust the volume. The PCR reaction was conducted under the following conditions, 5 min at 94 °C, followed by 35 cycles at 94 °C for 30 s, 54 °C for 30 s, and 72 °C for 90 s, with final extension for 10 min at 72 °C. Amplified PCR products were analyzed by agarose gel (1%) electrophoresis. After gel extraction, the products were purified by SanPrep pillar PCR purification kit (Sangon Biotech Co., Ltd.). Finally, the PCR products of strain GYL6 were sequenced on Illumina Miseq platform (Illumina Inc., San Diego, CA, USA) provided by Sangon Biotech Co., Ltd. The ribosomal sequences obtained were compared with those released in GenBank (National Center for Biotechnology Information, Bethesda, MD, USA) database using the Nucleotide Basic Local Alignment Search Tool program.

### 2.7. In vitro Digestibility

The in vitro digestibility of dry matter and digestible energy of FSBM and SBM were assayed with a computer-controlled simulated digestion system (CCSDS), which was developed and designed by Zhao et al. and Chen et al. [30,31] to simulate the gastrointestinal digestion system of monogastric animals. The machine automatically finishes the in vitro digestion process, including the gastric, upper- and lower-intestinal digestion, when the procedural instructions are followed. In brief, 2 g of FSBM or SBM and 20 mL simulated gastric fluid (1500 U/mL pepsin, pH 2.0) was placed in the dialysis tubing within the digestion chamber, and then the gastric buffer solution (pH 2.0, 41 °C) was pumped (120 mL/min) into the digestion chambers and circulated for 4 h. After gastric digestion, the upper-intestinal digestion was performed, 2 mL of concentrated simulated small-intestinal fluid (401.46 U/mL amylase, 49.28 U/mL trypsin, 11.31 U/mL chymotrypsin, pH 6.50) was injected into the digestion chamber, followed by injection of the upper-small-intestinal buffer solution (120 mL/min; pH 6.50, 41 °C) and circulation for 7.5 h. Finally, the lower-small-intestinal buffer solution (pH 8.0, 41 °C) was pumped into the digestion chambers and circulated for 7.5 h. During in vitro digestion, a temperature of 41 °C was maintained in a shaking incubator set to 180 rpm/min, to allow the sample and digestive fluid to mix adequately. After the simulated digestion was completed, the undigested residues were transferred to a preweighed vessel and dried overnight at 65 °C and then at 105 °C for 5 h until a constant weight was achieved. The vitro digestibility of dry matter and the digestible energy were calculated with the following equations:

In digestibility of dry matter (%) = (initial dry matter content − residual dry matter content) × 100%/initial dry matter content

In vitro digestible energy (MJ/kg) = (initial dry matter gross energy − residual dry matter gross energy)/(initial dry matter content × 1000)

### 2.8. Safety Assessment of the Candidate Strain

In the preliminary experiment, the candidate strain showed no harmful effect on the health of mice, so an acute oral toxicity test was performed with a maximal tolerated dose. Twenty healthy specific-pathogen-free (SPF) grade male C57BL/6J mice (7–8 weeks old) were obtained from Sibeifu (Beijing, China) Biotechnology Co., Ltd. (Beijing, China), certificate of conformity no. SCXK (Jing) 2016–0002). The mice were randomly divided into two groups based on body weight and housed five per cage. The room for mouse growth was maintained at a temperature of 23 ± 2 °C, a relative humidity of 50–55%, and a 12 h light-dark cycle. The mice were fed ad libitum and had free access to drinking water. After acclimation for 7 days, the mice in the treatment group were administered 0.4 mL of bacterial suspension (1.0 × 10^9^ CFU/mL) by oral gavage daily for 7 consecutive days, which was approximately equivalent to 2.0 × 10^10^ CFU per kg bodyweight, and the control group of mice was administered the same amount of physiological saline. After gavage, clinical observations were carried out and recorded daily, including any changes in skin, eyes, or fur, the occurrence of secretions or excretions, and autonomic activity, such as lacrimation, piloerection, and unusual respiratory patterns. Two weeks after gavage, the mice were killed by cervical dislocation. Their spleens, livers, and intestinal tracts were subjected to necropsy and histopathological examination. The spleens, livers, and hearts were also immediately weighed and the organ ratios calculated. The present study was approved by the Animal Ethics Committee of Chinese Academy of Agricultural Science (code number: AEC-CAAS-20190721; approval date: 21 July 2019) and was conducted in accordance with the principles outlined in the National Institutes of Health (NIH) Guide for the Care and Use of Laboratory Animals.

### 2.9. Statistical Analysis

All statistical analyses were performed with the SPSS 19.0 software (SPSS Inc., Chicago, IL, USA). The data on crude protein, TCA-SP, and antigenic proteins were analyzed with one-way ANOVA and Duncan’s post-hoc test. The comparison of two means was tested using Student’s *t*-test. Difference were considered significant at *p* < 0.05.

## 3. Results

### 3.1. Screening Strains for SBM Fermentation

In a preliminary experiment, six candidate strains were successfully isolated from the rumen of cow on defatted milk agar plates for further screening. As shown in Table 1, the results of rescreening showed that five strains (GYL2, GYL3, GYL5, GYL6 and BSD2) produced transparent zones with greater diameters on the glycinin agar plates than the other strains, indicating their greater ability to degrade the glycinin. The transparent zone diameters of all strains except GYL1 and GYL4 on the β-conglycinin agar plates, indicated their capacity to degrade β-conglycinin. GYL4, GYL5, GYL6, and 21237 exhibited amylase activity on the starch agar plates, whereas of these eight strains, only GYL5 and GYL6 displayed xylanase activity. Strain GYL6 showed higher hydrolysis transparent zone diameter/colony diameter (HD/CD) ratios on these kinds of agar plates than GYL5. Therefore, strain GYL6 was selected as the candidate strain for further study. To confirm the suitable strain, solid state fermentation was conducted to verify the results of the agar plate analyses.

Table 2 shows the effects of fermentation with all the strains used in this study on the allergenic protein, crude protein, and TCA-SP contents in SBM. ELISAs showed that the maximal degradation of glycinin and β-conglycinin was reduced from 179.43 mg/g to 13.78 mg/g and from 135.83 mg/g to 20.30 mg/g, respectively, after fermentation with strain GYL6. Solid state fermentation with GYL2, GYL3, BSD2, and GYL6 for 24 h increased the content of TCA-SP in FSBM to 18.05%, 18.53%, 17.24%, 21.39%, respectively (*p* < 0.05). Notably, the content of TCA-SP was approximately 11-fold higher in FSBM than in SBM, after fermentation with GYL6. Fermentation with strain GYL6 increased the contents of TCA-SP and crude protein significantly more than fermentation with other strains (*p* < 0.05).

**Figure 1:**
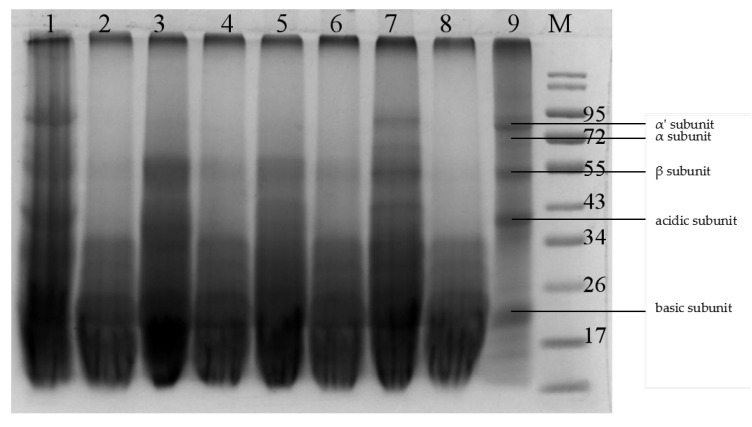
Protein electrophoresis analysis of SBM and FSBM produced by different strains. Lane 1, GYL1 fermented SBM; lane 2, GYL2 fermented SBM; lane 3, CICC21237 fermented SBM; lane 4, GYL3 fermented SBM; lane 5, GYL4 fermented SBM; lane 6, BSD2 fermented SBM; lane 7, GYL5 fermented SBM; lane 8, GYL6 fermented SBM; lane 9, unfermented SBM; M, protein molecular weight marker.

SDS-PAGE was conducted to further analyze the effects of fermentation with different bacterial strains on the SBM protein profile. As shown in Figure 1, SBM displayed all the subunit bands of the SBM antigenic protein (Figure 1, lane 9), including three subunits (α, α′, β) of β-conglycinin, and the two subunits (acidic and basic) of glycinin. fermentation with gyl6 successfully degraded the α, α′, and β subunits of β-conglycinin and the acidic subunit of glycinin (Figure 1, line 8). Most of the macromolecular proteins in SBM was below 34 kDa after fermentation with GYL6. By contrast, other strains, such as GYL1, GYL4, GYL5, and 21237 (Figure 1, lane 1, lane 5, lane 7, and lane 3, respectively), had weaker abilities to reduce content of the antigenic proteins in SBM, as illustrated by the ELISA analysis. Therefore, strain GYL6 was used in subsequent experiments.

### 3.2. Identification of Candidate Strain

The morphological and biochemical characteristics of GYL 6 were compared with other reference *B.amyloliquefaciens* reported by other research (Table 3), which suggested that the strain GYL 6 belonged to *B. amyloliquefaciens.* The full 16S rRNA sequence (Figure S1) of strain GYL6 shared 100% identity with *B. amyloliquefaciens* strain SX 16 NA (GenBank accession MT052665.1). Meanwhile, the complete gyrase B (gyrB) sequence (Figure S2) of strain GYL6 shared 100% identity with that of *B. amyloliquefaciens* ZJU1 (GenBank accession CP041691.1). Therefore, based on the above characteristics, strain GYL6 was identified as *B. amyloliquefaciens*.

### 3.3. Effect of Fermentation with B. amyloliquefaciens on the Amino Acid Composition of SBM

Table 4 shows the total amino acids detected in SBM and FSBM. The total amino acids content increased by 6.57% after fermentation with *B. amyloliquefaciens* for 24 h. The contents of some essential amino acids, including valine, methionine, phenylalanine, and histidine, were significantly increased in FSBM by 10.68%, 14.71%, 13.31%, and 16.13%, respectively. Among the nonessential amino acids, the contents of alanine, proline, cysteine, and glutamic in FSBM increased by 10.33%, 14.81%, 16.67%, and 20.21%, respectively, compared with those in unfermented SBM. However, the contents of arginine and serine were significantly lower in FSBM (*p* < 0.05).

### 3.4. Effect of Fermentation with B. amyloliquefaciens on In Vitro Digestibility of SBM

**Figure 2:**
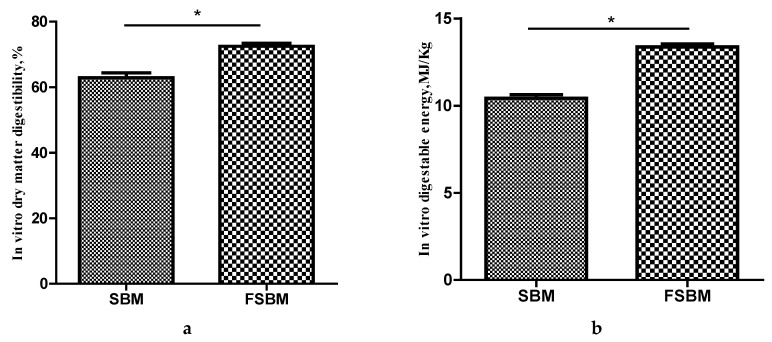
In vitro digestibility of SBM and FSBM. (**a**) In vitro dry matter digestibility of SBM and FSBM; (**b**) In vitro digestible energy of SBM and FSBM. * indicates significant differences at *p* < 0.05.

The results for the in vitro digestibility of SBM and FSBM are presented in Figure 2. Both the in vitro dry matter digestibility and the digestible energy increased significantly from 62.91% to 72.52% and from 10.42 MJ/kg to 13.37 MJ/kg (dry matter basis), respectively, after fermentation with *B. amyloliquefaciens* for 24 h (*p* < 0.05).

### 3.5. Effect of Administration of B. amyloliquefaciens on Bodyweight and Organ Indices of Mice

After the oral administration of *B. amyloliquefaciens* to mice for 7 days and their observation for 14 days, there were no deaths in either the control group or experimental group. No obvious symptoms of toxicity were detected in skin, fur, or eyes. No changes in other physiological activities were observed immediately after administration of *B. amyloliquefaciens* or during the posttreatment period in two groups. Neither of the mice showed any visible abnormality of the visceral organs of the mice during necropsy. All the mice grew well. As shown in Table 5, the final weight of experimental group slightly higher than that of control group, but not significantly so at *p* < 0.05 level. The organ indices of the mice, including of the heart, liver, and spleen, did not differ significantly between the control and treatment groups (*p* > 0.05).

### 3.6. Effect of Oral Administration of B. amyloliquefaciens on Intestinal Morphological Structure and Hispathology of Mice

Table 6 shows the villus height (VH), crypt depth (CD), and VH/CD ratio in the duodenum, jejunum, and ileum of the treated mice. The treatment group had higher VH in the duodenum and ileum, and a higher VH/CD ratio in the jejunum and ileum than the control group (*p* < 0.05). There were no significant differences in CD in the duodenum or ileum between the two treatment groups.

**Figure 3:**
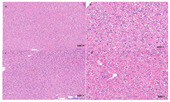
Pathological sections of liver tissues from mice. (**a**) control group(magnification 100×); (**b**) control group(magnification 400×); (**c**) experimental group(magnification 100×); (**d**) experimental group(magnification 400×).

**Figure 4:**
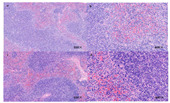
Pathological sections of spleen tissues from mice. (**a**) control group (magnification 100×); (**b**) control group(magnification 400×); (**c**) experimental group(magnification 100×); (**d**) experimental group(magnification 400×).

Histopathological examination of the tissues of the two treatment groups showed no visible pathological changes in the livers or spleens of the mice, and no abnormal macroscopic structures of the livers or spleens. There was no swelling, necrosis, degeneration, inflammatory changes, or cell infiltration in the pathological sections of the livers or spleens, (Figure 3 and Figure 4), respectively.

## 4. Discussion

Fermented feed has the advantages of improving the feed quality and gut ecology of animals compared with supplementation with feed with additives, such as probiotics, prebiotics, synbiotics, and enzymes [34]. The kind of microorganisms used in this fermentation is one of the major factors affecting the nutritional value of feed and feed ingredients. *Bacillus* ssp. are the predominant fermentative microorganisms used to ferment feed. In previous studies, *Bacillus* ssp. were used to ferment SBM from traditional fermented soy food [22,23], and aquatic animals, such as shrimp guts [35] and grass carp intestine [24], respectively. They were screened with soymilk, xylan, and starch agar plate methods. However, the ability of the target strains to degrade glycinin and β-conglycinin could not be determined indirectly with these agar plate tests. In the present study, glycinin and β-conglycinin were extracted from SBM and used as the sole substrates upon which to culture the candidate strains. The abilities of the candidate strains to degrade the antigenic proteins were compared directly by measuring the diameters of the transparent zones on the culture plates. Among these eight isolates, strain GYL6 showed higher proteinase, xylanase, and amylase activities than other seven strains on agar plates. The results of solid state fermentation with these eight strains confirmed that strain GYL6 was the most efficient strain. It eliminated most glycinin and β-conglycinin from the SBM, and significantly increased its content of crude protein and TCA-SP. These results were consistent with the results of agar plate test method. These findings show that plate tests are an effective and convenient method to screen suitable strains in future studies.

It is recommended that gyr B gene sequences be used, as well as 16S rRNA gene sequences, to identify bacterial strains, because the high genetic similarity of *Bacillus* spp. make them difficult to discriminate. A previous study demonstrated that the gyrB gene has a faster rate of molecular evolution than 16S rRNA and more precisely identifies bacterial strains [36]. In the current study, strain GYL6 was identified as *B. amyloliquefaciens* using both 16S rRNA and gyrB sequences.

This study clearly demonstrates that 92.32% of glycinin and 85.05% of β-conglycinin were removed after fermentation with *B. amyloliquefaciens* for 24 h. These results are similar to those reported by Zhang et al., who found that 84.77% of glycinin and 87.07% of β-conglycinin were degraded after fermentation with *B. subtilis* BS12 for 24 h [22]. By contrast, only 42% of glycinin and 70% of β-conglycinin were eliminated by another strain of *B. subtilis* [16]. Under optimum fermentation conditions (temperature 40 °C, time 48 h and 4% inoculum (*w*/*v*)), the glycinin in SBM fermented with *B*. *sanfensis* or *B*. *stratosphericus* decreased by only 62.0% or 41.1%, respectively [35]. These differences in the degradation of the antigenic proteins in SBM indicates that the *B*. *amyloliquefaciens* isolated in the present study has a stronger capacity for protein hydrolysis. Recently, we found that *B*. *amyloliquefaciens* secretes many extracellular hydrolytic enzymes during its fermentation of SBM, including not only serine protease and acid protease, but also endoglucanase, acetylxylan esterase, and catalase, which broke down cell-wall polymers and facilitated the degradation of antigenic protein (unpublished data). The reduction of antigenic soybean proteins increases the digestibility of nutrients and improves the growth performance of animals [9,37]. Previous studies have demonstrated that FSM enhances intestinal barrier function [38,39], attenuates the intestinal mucosal immune response [9], and reduces the incidence of diarrhea, which are related to the reduction in the antigenic proteins of SBM.

Several reports had demonstrated that fermentation with different *Bacillus* spp. substantially increases the crude protein content of SBM [17,18,40]. In the present study, crude protein increased significantly by 17.54% after fermentation with *B. amyloliquefaciens* for 24 h. The increase in crude protein may be attributable to the loss of carbohydrates, which might be used as substrates in the growth and reproduction of bacteria. At the same time, the content of TCA-SP, which consists of small peptides with 2–20 residues, was approximately 11-fold higher in FSBM than in SBM. A previous study demonstrated that dipeptides and tripeptides are directly absorbed by the animal intestinal tract, and that amino acids in the form of small peptides are transported more rapidly than their constituent amino acids in free form [22]. Our analysis of the in vitro digestibility suggested that the fermentation of SBM with *B. amyloliquefaciens* enhanced its nutritional value and it was digested easily by digestive enzymes.

In present study, SBM fermented with *B. amyloliquefaciens* significantly increased the total amino acids by 6.57% and changed its amino acids composition. The essential amino acids, including valine, methionine, phenylalanine, and histidine, were 10–17% higher in FSBM than in SBM. These results are partly consistent with the findings of Medeiros et al., who reported that the contents of lysine, valine, histidine, and phenylalanine increased by 15–19% in FSBM fermented with *B. amyloliquefaciens*. Among these essential amino acids, methionine, the first limiting amino acid in the corn-soybean meal diet, significantly influences the energy metabolism, immune function [41] and growth performance of animals [42]. Valine is a functionally essential branched-chain amino acid and the fourth limiting amino acid, which also plays an important role in improving growth performance and enhancing the immunity of animals [43]. Therefore, these increases in some essential amino acids could reduce the currently necessary supplementation of animal feed with synthetic amino acids. However, the amino acid composition of SBM is altered differently when it was fermented with different microorganisms. In the present study, we found that the lysine content remained unchanged, whereas methionine increased in FSBM. These results are identical to those reported by Chi and Cho [18], but contrary to findings of Gao et al. [35], who demonstrated that the content of methionine was more than two fold higher in FSBM than in SBM. Hassaana et al. reported that except for serine and histidine, all the amino acids in SBM increased significantly after fermentation with *Saccharomyces cerevisiae* [44], whereas in another study, the essential amino acid profile of SBM was unchanged after fermentation with *A. oryzae* GB-107 [45]. The discrepancies in these finding may be attributable to the proteinase profiles and secretion abilities of the microorganism used [15]. These findings also indicate that the suitable microorganism for fermentation must be selected in accordance with the nutritional requirements of final fermented feed product.

Today, many researchers and companies use strains from different sources to directly ferment feed, without considering the potential risks of the isolated strains. Previous studies have reported that some *Bacillus* spp., including *B. cereus*, *B. coagulans*, and *B. weihenstephanesis*, produce both enterotoxins and emetic toxins, which are recognized as the main causes of food poisoning and other non-gastrointestinal infections [46]. Therefore, it is important to comprehensively evaluate the safety of a bacterial strain before it is incorporated into animal feed [26].In vitro and in vivo method are the main approach to evaluate the safety of probiotics. In vivo method includes detecting the known toxin gene, using cultured cell line to evaluate adhesion, invasion, and cytotoxicity of probiotics [47,48]. In vivo studies are the preferred approach to substantiate in vitro analysis of safety [49]. However, no specific guidelines are currently in place for assessing probiotics, but acute oral toxicity testing is the fundamental assay for assessing the safety of novel microbial strains intended as feed additives or for use in feed production, and has been applied in many studies [14,50]. For an acute toxicity study, mice were chosen because they are considered the most sensitive laboratory animals [47]. Based on the results of our preliminary experiment, acute oral toxicity testing was performed by intragastrically administering *B. amyloliquefaciens* to mice at a maximal dose of 2.0 × 10^10^ CFU/kg BW for 7 consecutive days in this study. At the end of this experiment, all the mice were healthy, with no noticeable abnormal behavior or changes in activities. There were no significant changes in the relative weight of the major organs of mice. Relative organ weight has long been recognized as an important indicator of chemical organ damage [46,51]. Hyperplastic changes, congestion, edema, and visceral atrophy also reflect the damage caused by poisonous substances [51]. In the present study, there were no differences in the relative organ weights or morphological structures of the tissues after the administration of *B. amyloliquefaciens*, indicating that the strain had no adverse effects on the health status of mice and can be considered safe for fermented feed production.

## 5. Conclusions

In conclusion, a highly efficient *B. amyloliquefaciens* strain was successfully isolated from the rumen of a cow with plate tests and fermentation. Fermentation with *B. amyloliquefaciens* effectively degraded the glycinin and β-conglycinin in SBM, significantly improved the crude protein content and acid soluble protein concentration, and increased the total amino acid content. Our results also show that the intragastric administration of *B. amyloliquefaciens* at the maximal dose had no adverse effects on animal health. These results indicate that the *B. amyloliquefaciens* strain isolated in this study is safe for animal use and can be widely applied to SBM fermentation.

## Figures and Tables

**Table 1:** Size of the transparent zone formed by the actions of enzyme of candidate strains.

Strain	Protease ^a^			Protease ^b^			Amylase			Xylanase		
	HD ^c^ (cm)	CD ^d^ (cm)	HD/CD	HD (cm)	CD (cm)	HD/CD	HD (cm)	CD (cm)	HD/CD	HD (cm)	CD (cm)	HD/CD
GYL6	2.70	1.00	2.70	2.70	1.10	2.45	2.00	1.10	1.82	1.73	1.23	1.41
GYL5	2.83	2.30	1.21	2.43	1.47	1.66	2.73	2.43	1.12	2.73	2.42	1.12
21237	-	-	-	1.13	0.57	2.00	1.27	0.67	1.9	-	-	-
BSD2	1.70	0.60	2.83	1.47	0.60	2.44	-	-	-	-	-	-
GYL4	-	-	-	-	-	-	1.13	0.87	1.31	-	-	-
GYL3	1.67	0.53	3.13	1.43	0.53	2.53	-	-	-	-	-	-
GYL2	1.63	0.53	3.06	1.37	0.53	2.56	-	-	-	-	-	-
GYL1	-	-	-	-	-	-	-	-	-	-	-	-

^a^ Protease represents the ability of the strain to degrade glycinin; ^b^ Protease represents the ability of the strain to degrade β-conglycinin; ^c^ HD represents hydrolysis transparent zone diameter; ^d^ CD represents colony diameter. - represents no detection.

**Table 2:** Effects of fermentation with different candidate strains on the contents of TCA-SP, antigenic protein, and crude protein in SBM.

Items	TCA-SP, %	Crude Protein, %	Glycinin		β-Conglycinin	
			Content, mg/g	Degradation ^1^, %	Content, mg/g	Degradation, %
SBM	1.95 ^g^	46.92 ^c^	179.43 ^a^	-	135.83 ^a^	-
GYL6 ^2^	21.39 ^a^	55.15 ^a^	13.78 ^e^	92.32	20.30 ^e^	85.05
GYL5	10.12 ^d^	54.55 ^a^^,^^b^	131.95 ^b^	26.46	56.46 ^b^	58.43
21237	8.34 ^e^	53.05 ^b^	147.83 ^b^	17.61	61.81 ^b^	54.49
BSD2	17.24 ^b^	54.29 ^ab^	32.92 ^d^	81.65	31.55 ^d^	76.77
GYL4	11.78 ^c^	53.95 ^b^	116.40 ^c^	35.13	51.52 ^c^	62.07
GYL3	18.53 ^b^	53.45 ^b^	29.89 ^d^	83.34	28.89 ^d^	78.73
GYL2	18.05 ^b^	53.74 ^b^	27.21 ^d^	84.83	27.65 ^d^	79.64
GYL1	6.77 ^f^	53.64 ^b^	139.24 ^b^	22.39	58.95 ^b^	56.60
SEM	1.27	0.52	7.89	-	7.12	-
*p*-value	<0.01	<0.01	<0.01	-	<0.01	-

^1^ Degradation rate = (antigenic protein content in SBM -antigenic protein content in FSBM)/antigenic protein content in SBM × 100%; ^2^ GYL (no.) means FSBM produced by this strain. Different subscript letters indicate significant differences in the column at *p* < 0.05.

**Table 3:** The morphological and biochemical characteristics of GYL 6 and other *B. amyloliquefaciens.*

Items	GYL6	*B. amyloliquefaciens S20*^a^ [32]	*B. amyloliquefaciens LN2*^b^ [33]
Cell shape	Rod	Rod	Rod
Gram staining	+	+	+
D-Glucose	+	+	+
D-Xylose	+	+	+
L-Rhamnose	−	No detected	−
D-Mannitol	+	+	+
L-Arabinose	+	No detected	+
Maltose	+	+	+
Sucrose	+	+	+
Lactose	+	No detected	+
Malonate	−	No detected	No detected
Starch	+	+	+
Citrate	+	+	+
Nitrate reduction test	+	+	No detected
VP-reaction	−	+	No detected

“+” represents positive reaction; “−” represents negative reaction; ^a^ Chen et al.; ^b^ Lee et al.

**Table 4:** Amino acid composition of SBM fermented for 24 h with *B. amyloliquefaciens.*

Items	SBM	FSBM	SEM	*p*-Value
Essential amino acid				
Arg	3.36	2.95	0.01	<0.01
His	1.24	1.44	0.01	<0.01
Lys	3.10	3.13	0.02	0.21
Met	0.68	0.78	0.03	0.03
Thr	1.83	1.90	0.02	0.04
Ile	2.29	2.28	0.03	0.77
Leu	3.76	3.82	0.06	0.34
Phe	2.48	2.81	0.02	<0.01
Val	2.34	2.59	0.03	<0.01
Nonessential amino acid				
Cys	0.66	0.77	0.01	<0.01
Gly	2.04	2.19	0.01	<0.01
Ala	2.13	2.35	0.06	0.02
Tyr	1.55	1.73	0.06	0.06
Asp	5.51	5.67	0.01	<0.01
Ser	2.41	2.13	0.02	<0.01
Pro	2.43	2.79	0.03	<0.01
Glu	8.46	10.17	0.16	<0.01
Total	46.40	49.45	0.47	0.02

**Table 5:** Effect of administration of *B. amyloliquefaciens* on bodyweight and organ indices of mice.

Items	Body Weight		Organ Index of Mice (%)			Mortality Rate (%)
	Initial Weight (g)	Final Weight (g)	Heart	Liver	Spleen	
control group	23.20	26.07	0.54	4.26	0.31	0
treatment group	23.20	26.73	0.53	4.15	0.32	0
SEM	0.21	0.30	0.01	0.05	0.01	-
*p*-value	1.00	0.16	0.45	0.20	0.47	-

**Table 6:** Effect of oral administration with *B. amyloliquefaciens* on the intestinal morphological structures of mice.

Items		Control Group	Experimental Group	SEM	*p*-Value
Duodenum	Villus height (VH)	381.09	438.77	12.06	0.01
	Crypt depth (CD)	72.80	85.85	8.05	0.32
	VH: CD ratio	5.57	5.26	0.54	0.78
Jejunum	Villus height (VH)	261.17	280.91	4.01	0.23
	Crypt depth (CD)	94.91	79.27	5.71	0.04
	VH: CD ratio	2.75	3.61	0.29	0.03
Ileum	Villus height (VH)	225.42	303.00	5.4	<0.01
	Crypt depth (CD)	75.66	83.29	3.28	0.27
	VH: CD ratio	2.97	3.64	0.11	<0.01

## Supplementary Materials

The following are available online at https://www.mdpi.com/2076-2615/10/7/1144/s1, Figure S1: The 16S rDNA sequences amplified from *B. amyloliquefaciens*, Figure S2: The gyrase B sequences amplified from B. amyloliquefaciens.
