# Supplementary material for: Isolation of a Highly Efficient Antigenic-Protein-Degrading Bacillus amyloliquefaciens and Assessment of Its Safety
Source: Animals (Basel). 2020 Jul 6;10(7):1144. doi: 10.3390/ani10071144 (PMC7401624; doi:10.3390/ani10071144)
Supplement: Supplementary file 1 [file animals-10-01144-s001.pdf]

Supplementary Materials:

Figure S1. The 16S rDNA sequences amplified from *B. amyloliquefaciens*.

1 ACAAACTCTCGTGGTGTGAC GGGCGGTGTGTACAAGGCCC GGGAACGTATTCACCGCGGA
61 TGCTGATCCGCGATTACTAG CGATTCCAGCTTCACGCAGT CGAGTTGCAGACTGCGATCC
121 GAACTGAGAACAGATTTGTG GGATTGGCTTAACCTCGCGG TTTCGCTGCCCTTTGTTCTG
181 CCCATTGTAGCACGTGTGTA GCCCAGGTCATAAGGGGCAT GATGATTTGACGTCATCCCC
241 ACCTTCCTCCGGTTTGTCAC CGGCAGTCACCTTAGAGTGC CCAACTGAATGCTGGCAACT
301 AAGATCAAGGGTTGCGCTCG TTGCGGGACTTAACCCAACA TCTCACGACACGAGCTGACG
361 ACAACCATGCACCACCTGTC ACTCTGCCCCCGAAGGGGAC GTCCTATCTCTAGGATTGTC
421 AGAGGATGTCAAGACCTGGT AAGGTTCTTCGCGTTGCTTC GAATTAAACCACATGCTCCA
481 CCGCTTGTGCGGGCCCCCGT CAATTCCTTTGAGTTTCAGT CTTGCGACCGTACTCCCCAG
541 GCGGAGTGCTTAATGCGTTA GCTGCAGCACTAAGGGGCGG AAACCCCCTAACACTTAGCA
601 CTCATCGTTTACGGCGTGGA CTACCAGGGTATCTAATCCT GTTCGCTCCCCACGCTTTCG
661 CTCCTCAGCGTCAGTTACAG ACCAGAGAGTCGCCTTCGCC ACTGGTGTTCCTCCACATCT
721 CTACGCATTTCACCGCTACA CGTGGAATTCCACTCTCCTC TTCTGCACTCAAGTTCCCCA
781 GTTTCCAATGACCCTCCCCG GTTGAGCCGGGGGCTTTCAC ATCAGACTTAAGAAACCGCC
841 TGCGAGCCCTTTACGCCCAA TAATTCCGGACAACGCTTGC CACCTACGTATTACCGCGGC
901 TGCTGGCACGTAGTTAGCCG TGGCTTTCTGGTTAGGTACC GTCAAGGTGCCGCCCTATTT
961 GAACGGCACTTGTTCTTCCC TAACAACAGAGCTTTACGAT CCGAAAACCTTCATCACTC
1021 ACGCGGCGTTGCTCCGTCAG ACTTTCGTCCATTGCGGAAG ATTCCCTACTGCTGCCTCCC
1081 GTAGGAGTCTGGGCCGTGTC TCAGTCCCAGTGTGGCCGAT CACCCTCTCAGGTCGGCTAC
1141 GCATCGTCGCCTTGGTGAGC CGTTACCTCACCAACTAGCT AATGCGCCGCGGGTCCATCT
1201 GTAAGTGGTAGCCGAAGCCA CCTTTTATGTCTGAACCATG CGGTTCAAACAAGCATCCGG
1261 TATTAGCCCCGGTTTCCCGG AGTTATCCCAGTCTTACAGG CAGGTTACCCACGTGTTACT
1321 CACCCGTCCGCCGCTAACAT CAGGGAGCAA

Figure S2. The gyrase B sequences amplified from B. amyloliquefaciens.

1 ACGAGCGCCAGCAGGTTGAT TCCGAAAGACGTCTGCAGGG CCGTTTGTTTGTACAGGTTA
61 TTCAAAATGACGTGAGCATT GGCGTCACGGCGGATCTCAA TGACGATTCTCATTCCGTTA
121 CGGTCGGATTCGTCTCGCAG GTCGGTAATTCCTTCGATTT TTTTGTCCCGGACAAGATCT
181 GCGATTTTTTCAATTAATCT CGCTTTGTTCACCTGATAAG GAAGTTCCGTAACAATAATT
241 CTTTCTTTTCCTGATGATGT CTCTTCGATTTCAGCCTTAG CCCGGATTGTGATTGATCCC
301 CGTCCGGATTCATATGCCTT GCGGATGCCGCTCCGGCCCA AAATCTGACCAGCAGTCGGA
361 AAATCCGGGCCCGGGATGTA TTCCATCAGCTCCTGGTTTG TAATCTCAGGATTCTCACTT
421 ACGGCAAGCACGCCTTCAAT GACTTCTCCAAGCTGATGGG GAGGAATGTTTGTCGCCATT
481 CCGACCGCAATACCGGCAGC CCCGTTTACGAGCAGATTCG GAAATCTCGAAGGCATGACG
541 GCAGGCTCTCTTTCTGAACC GTCATAGTTATCTTGATAGT CAATCGTGTCTTTCGTAATG
601 TCACGCAGAATTTCCATTGC GATTTTTGACATTCTCGCTT CTGTGTAACGCATCGCGGCC
661 GCTGAGTCGCCGTCAACCGA ACCGAAGTTGCCGTGTCCGT CAACAAGCATGTAGCGGTAG
721 TTAAAATCCTGCGCCATTCT GACCATTGATTCGTAAACCG CTGAGTCACCGTGCGGGTGG
781 TACTTACCGATAACTTCACC GACGATACGGGCAGATTTTT TATATGGTTTGTCACTGGTC
841 ATGCCTAAATCATTCATTGC GTACAAAATCCGTCTGTGAA CCGGCTTCAGACCGTCACGC
901 ACATCCGGAAGCGCCCGGGA TACGATAACGCTC
